# Efficacy of Upadacitinib Retreatment or Dose Escalation After Loss of Response in Ulcerative Colitis: Data From the Open-Label Extension of the U-ACTIVATE Study

**DOI:** 10.1016/j.gastha.2026.100969

**Published:** 2026-04-13

**Authors:** Remo Panaccione, Jean-Frédéric Colombel, Marla Dubinsky, Christopher Ma, Michelle Kujawski, Erica Cheng, Elena Dubcenco, Sina Ogholikhan, Elena Marced Barrachina, Tadakazu Hisamatsu

**Affiliations:** 1 Division of Gastroenterology and Hepatology, University of Calgary, Calgary, Alberta, Canada; 2 Division of Gastroenterology and Hepatology, Icahn School of Medicine at Mount Sinai, New York, New York; 3 AbbVie Inc., North Chicago, Illinois; 4 Department of Gastroenterology and Hepatology, Kyorin University School of Medicine, Tokyo, Japan

**Keywords:** JAK Inhibitor, Retreatment, Recapture, Treatment Interruption

## Abstract

**Background and Aims:**

Upadacitinib (UPA) is a Janus kinase inhibitor approved for moderately to severely active ulcerative colitis (UC). Patients may discontinue and then restart UPA treatment or require dose adjustment; however, the impact on overall efficacy of treatment interruption or dose escalation is not fully characterized. This analysis of the U-ACTIVATE (NCT03006068) open-label extension (OLE) study evaluated the efficacy of UPA retreatment after UPA withdrawal or dose escalation during UPA maintenance following a loss of response in patients with UC.

**Methods:**

Patients who responded to 8 weeks of UPA 45 mg induction therapy were rerandomized to placebo, UPA 15 mg (UPA15), or 30 mg (UPA30) in the 52-week U-ACHIEVE (NCT02819635) maintenance study. Patients who subsequently lost response on placebo during maintenance could enter the U-ACTIVATE OLE and were retreated with UPA at a 15 mg dose (UPA retreatment group); patients who lost response on maintenance UPA15 continued UPA15 during the OLE. Patients who lost response on UPA15 during the OLE and met prespecified criteria were escalated to UPA30. Clinical and endoscopic remissions were assessed through week 144. Safety was not assessed in this analysis but has been reported previously.

**Results:**

The UPA retreatment group enrolled 110 patients; 57 remained on UPA15 (UPA15OLE→UPA15) and 47 were escalated to UPA30 (UPA15OLE→UPA30) by week 144. A total of 39 patients lost response on maintenance UPA15 and entered the OLE on UPA30 (UPA15Maint→UPA30). At week 144, the following proportion of patients achieved clinical remission per adapted Mayo score and endoscopic remission, respectively: UPA15OLE→UPA15: 76.3% and 33.3%, UPA15OLE→UPA30: 61.1% and 56.4%, and UPA15Maint→UPA30: 43.5% and 40.7%.

**Conclusion:**

In patients with UC, clinical and endoscopic efficacy could be recaptured following UPA retreatment or dose escalation in the U-ACTIVATE OLE.

## Introduction

Ulcerative colitis (UC) is a chronic inflammatory bowel disease characterized by periods of relapse and remission. Management of moderately to severely active UC often requires advanced therapies, including biologic and novel small-molecule agents, which may involve the induction of remission using higher drug doses followed by long-term maintenance regimens using lower doses.^1^^,^^2^ Due to the chronic nature of treatment, dose reduction or drug discontinuation has been explored as potential options for managing UC.^2^, ^3^, ^4^ The reasons for discontinuing or interrupting therapy for UC are multifactorial and include managing comorbidity (eg, surgery, infection), vaccination, wish for pregnancy, local insurance reimbursement limitations or to help reduce out-of-pocket patient costs, and a patient’s elective discontinuation based on perceived health improvement (which is not recommended).^5^^,^^6^ Alternatively, the treatment dose may be reduced due to perceived safety concerns or in specific patient populations, including those who are in long-term remission or those with a less severe disease history.^4^ The risks of treatment discontinuation are manifold, including relapse or complications,^2^^,^^5^^,^^6^ but patient-specific clinical outcomes of discontinuing and restarting UC treatment are yet to be fully understood.^6^

Upadacitinib (UPA) is an oral, reversible Janus kinase (JAK) inhibitor approved for adults with moderately to severely active UC.^7^, ^8^, ^9^ UPA met the standardized trial reporting recommendations in IBD – phase II recommendations of achieving clinical response and remission,^10^ in the U-ACHIEVE and U-ACCOMPLISH induction studies and the U-ACHIEVE maintenance study.^7^^,^^8^^,^^11^^,^^12^ U-ACTIVATE is a phase 3 open-label extension (OLE) study evaluating the long-term efficacy and safety of UPA in patients with moderately to severely active UC who enrolled in the U-ACHIEVE and U-ACCOMPLISH studies.^13^ An interim analysis of U-ACTIVATE demonstrated a favorable benefit-risk profile of both UPA 15 mg and UPA 30 mg.^13^ In this analysis of the U-ACTIVATE OLE, we assessed the efficacy of UPA retreatment in patients who lost response following UPA treatment withdrawal or maintenance on UPA 15 mg.

## Methods

### Study Design and Patient Population

The U-ACTIVATE (NCT03006068) OLE is an ongoing 288-week, phase 3 study involving adults with moderately to severely active UC who completed the U-ACHIEVE maintenance study. The study is being conducted at 307 centers across 43 countries and began on January 31, 2017. Details of the induction (U-ACHIEVE [phase 2b and phase 3; NCT02819635] and U-ACCOMPLISH [phase 3; NCT03653026]) and maintenance (U-ACHIEVE [phase 3; NCT02819635]) studies have been reported previously.^7^^,^^8^^,^^11^^,^^12^ An interim analysis of U-ACTIVATE through week 96 has also been previously described.^13^

**Figure 1:**
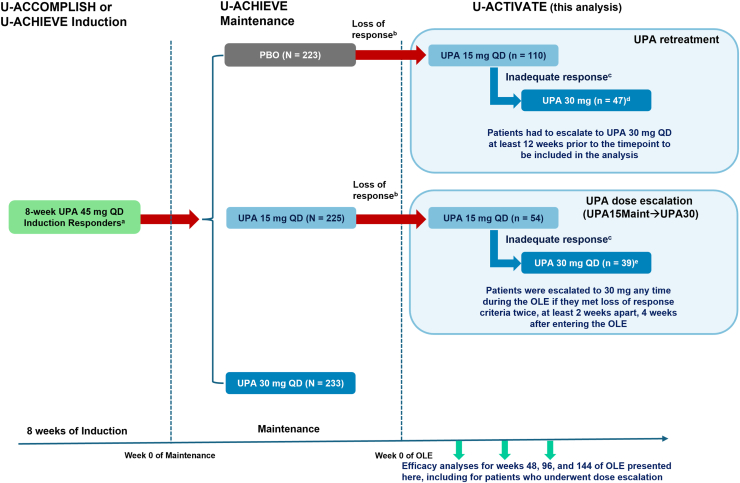
Study design schematic. ^a^Clinical response was defined as a decrease in adapted Mayo score of ≥2 points and ≥30% from baseline, plus a decrease in RBS of ≥1, or an absolute RBS of ≤1. ^b^Among patients with mean daily SFS and RBS <2.1 at maintenance week 0: an SFS and RBS each ≥1-point greater than the maintenance week 0 value on 2 consecutive visits ≥14 days apart. Among patients with SFS or RBS ≥2.1 at maintenance week 0: either an SFS or RBS ≥1-point greater than the maintenance week 0 value on 2 consecutive visits ≥14 days apart. ^c^Patients with an inadequate response (SFS + RBS that is unchanged or has increased from week 0 on 2 consecutive visits ≥7 days apart) to UPA 15 mg QD could be dose-escalated to UPA 30 mg QD between weeks 2 and 36 of the OLE. ^d^Of the 53 total patients escalated to UPA 30 mg QD, 6 (5.5%) patients were de-escalated to UPA15 mg QD and were not included in this analysis. ^e^Of the 43 total patients escalated to UPA 30 mg QD, 4 (9.3%) were de-escalated to UPA15 mg QD and were not included in this analysis. OLE, open-label extension; PBO, placebo; QD, once daily; RBS, rectal bleeding subscore; SFS, stool frequency subscore; UPA, upadacitinib.

The patient flow and populations for this analysis are shown in Figure 1. Patients who had a clinical response (per adapted Mayo score) after 8 weeks of UPA 45 mg in the 2 induction studies were rerandomized to receive UPA 15 mg, UPA 30 mg, or withdrawn to placebo for 52 weeks in the U-ACHIEVE maintenance study.^7^^,^^8^^,^^12^ Patients who subsequently lost response on placebo during the 52-week maintenance study could enter the U-ACTIVATE OLE and were treated with UPA 15 mg (UPA retreatment). Among these patients in the OLE, those who met the loss of response criteria were escalated to UPA 30 mg (UPA15OLE→UPA30). Patients who lost response to UPA 15 mg in U-ACHIEVE maintenance and entered the OLE continued on UPA 15 mg. These patients were dose escalated to UPA 30 mg (UPA15Maint→UPA30) if they met the loss of response criteria twice, at least 2 weeks apart, 4 weeks after entering the OLE.

Loss of response during maintenance was defined as an stool frequency score (SFS) and rectal bleeding score (RBS) each ≥1 point greater than the end-of-induction value (week 8 of induction) on 2 consecutive visits at least 14 days apart, or either an SFS or RBS ≥1 point greater than the end-of-induction value on 2 consecutive visits at least 14 days apart, associated with the presence of signs or symptoms of disease progression per investigator assessment. Loss of response during the OLE was defined as SFS > 1 and RBS > 0 on 2 consecutive visits, with at least 7 days apart between the 2 visits, or an endoscopic Mayo score = 2 or 3 based on the sites' local reading.

All patients provided written informed consent before enrollment in U-ACTIVATE, consistent with local regulations and standard operating procedures of the institutional review board. The study was conducted in accordance with the study protocol, International Conference on Harmonization guidelines, applicable regulations and guidelines governing clinical study conduct, and the ethical principles that have their origin in the Declaration of Helsinki. In Japan, after marketing approval for the treatment of UC, this study was conducted in compliance with the Ministerial on Good Post-marketing Study Practice. Ethics approval was obtained through central and local institutional review boards. All authors had access to relevant study data and reviewed and approved the final manuscript for publication.

### Efficacy Assessments

The proportion of patients achieving clinical remission per adapted and partial adapted Mayo score, endoscopic improvement, and endoscopic remission were assessed at the end of maintenance (week 0 of the OLE) and at weeks 48, 96, and 144 of the OLE. Clinical remission per adapted Mayo score was defined as an SFS ≤ 1 and not greater than baseline, RBS of 0, and endoscopy subscore ≤1 (without friability). Clinical remission per partial adapted Mayo score was defined as a decrease from baseline ≥1 points and ≥30% from baseline, and a decrease in RBS ≥ 1 or an absolute RBS ≤ 1. Endoscopic improvement was defined as an endoscopy subscore ≤1. Endoscopic remission was defined as an endoscopy subscore of 0.

### Endoscopy

Endoscopies were either full colonoscopies or flexible sigmoidoscopies depending on the extent of the disease at screening. Endoscopies were conducted every 48 weeks during the OLE with a window of ±14 days. The endoscopy was performed up to the segment where a clear demarcation of inflammation was observed between normal and inflamed mucosa and documented the distance from the anal verge. All endoscopies were reviewed by an independent primary central reader who was blinded to the patient’s clinical data, the site's endoscopy assessment and the patient’s therapy.

### Statistical Analyses

Efficacy analyses were conducted in the intent-to-treat analysis set, which is defined as all patients who received at least 1 dose of UPA. For demographics, baseline characteristics, and efficacy analyses, "baseline" was defined as the visit in which the first dose of study drug (UPA or placebo) was administered during the induction period. Summary statistics were provided for all efficacy variables at each visit based on observed data. These include the number of observations, mean, standard deviation, minimum, median, and maximum for continuous variables, and count and percent for discrete variables. The as-observed (AO) analysis was the primary analysis method for all binary and continuous end points. The AO analysis did not impute values for missing evaluations, and thus a patient who did not have an evaluation on a scheduled visit was excluded from the AO analysis for that visit. AO included all values collected in the study and included data after the first dose change. All statistical analyses were conducted with SAS (version 9.4 of the SAS system for Unix).

## Results

### Patient Population

A total of 681 patients in the induction studies had a clinical response to UPA 45 mg. Of these patients, 223 (32.7%) were rerandomized to placebo, 225 (33.0%) were rerandomized to UPA 15 mg during the U-ACHIEVE maintenance study. The remainder (n = 233, 34.2%) were rerandomized to UPA 30 mg and not included in this analysis (Figure 1). In total, 112/223 (50.2%) patients who were randomized to placebo during the maintenance study lost response (met loss of response criteria); 110/112 (98.2%) patients who lost response on placebo during maintenance subsequently entered the U-ACTIVATE OLE and were treated with UPA 15 mg starting at week 0 of the OLE. Among the 110 patients who entered the OLE after losing response on placebo during the maintenance study, 72 (65.5%) patients remained on UPA 15 mg and 32 (29.1%) patients were escalated to UPA 30 mg at week 48 (Supplementary Figure). Following dose escalation through week 48, 6 (5.5%) patients were de-escalated to UPA 15 mg and thus were not included in this analysis. The number of patients escalated to UPA 30 mg increased over time; 65 (59.1%) patients remained on UPA 15 mg and 39 (35.5%) patients were escalated to UPA 30 mg at week 96, while 57 (51.8%) patients remained on UPA 15 mg and 47 (42.7%) patients were escalated to UPA 30 mg at week 144. The median time to escalation to UPA 30 mg was 99 days (95% confidence interval: 68–218).

A total of 54/225 (24.0%) patients who were randomized to UPA 15 mg during the maintenance study lost response; 43/225 (19.1%) patients subsequently entered the U-ACTIVATE OLE on UPA 15 mg and were escalated to UPA 30 mg after 4 weeks upon meeting the loss of response criteria twice in those 4 weeks. Of these 43 patients, 4 (9.3%) were de-escalated to UPA 15 mg and thus were not included in this analysis. The decision to de-escalate was made at the discretion of the site health-care providers, and the reasons for de-escalation are not known.

### Patient Characteristics

At induction baseline, patient characteristics were balanced among those who experienced loss of response on placebo during maintenance vs those who did not experience loss of response (Table), with the following exceptions: patients with loss of response vs those without were more likely to have a history of inadequate response, loss of response, or intolerance to ≥1 biologic (Bio-IR; 57.1% vs 46.8%, respectively); prior exposure to anti–tumor necrosis factor (anti-TNF) therapy (54.5% vs 41.4%); or corticosteroid use at baseline (42.0% vs 33.3%). At week 144 of the OLE, patient characteristics were comparable among those who escalated to UPA 30 mg vs those who did not escalate (Supplementary Table), with the following exceptions: patients who underwent dose escalation vs those who did not were more likely to be Bio-IR (66.0% vs 49.1%, respectively), have prior exposure to anti-TNF therapy (67.9% vs 42.1%, respectively), and have corticosteroid use at baseline (49.1% vs 35.1%, respectively).

**Table:** Baseline Characteristics of Patients Who Lost Clinical Response vs Patients Who Did Not Lose Clinical Response During Maintenance

	Maintenance: placebo (N = 223)		Maintenance: UPA15 (N = 225)	
	Loss of response^a^ (n = 112)	No loss of response (n = 111)	Loss of response^a^ (n = 54)	No loss of response (n = 171)
Female, n (%)	47 (42.0)	53 (47.7)	19 (35.2)	60 (35.1)
Age, y, mean	42.2 (14.5)	42.7 (14.5)	39.0 (13.2)	42.5 (14.4)
Race, n (%)				
Asian	35 (31.3)	30 (27.0)	12 (22.2)	58 (33.9)
White	72 (64.3)	69 (62.2)	42 (77.8)	104 (60.8)
Other	5 (4.5)	12 (10.8)	0	9 (5.3)
Disease duration, y	8.0 (7.2)	8.8 (8.3)	7.8 (6.7)	8.3 (7.4)
Disease extent, n (%)				
Rectosigmoid	0	0	0	0
L-sided	61 (54.5)	56 (50.5)	24 (44.4)	78 (45.6)
Pancolitis	51 (45.5)	55 (49.5)	30 (55.6)	93 (54.4)
FCP, mg/L	3311 (5205)	3042 (4182)	3903 (5663)	3167 (5253)
hsCRP, mg/L	9.3 (16.2)	10.2 (15.9)	8.6 (12.7)	7.4 (10.8)
Bio-IR, n (%)	64 (57.1)	52 (46.8)	33 (61.1)	76 (44.4)
Prior exposure to biologic therapy for non-Bio-IR, n (%)	4 (8.3)	1 (1.7)	0	2 (2.1)
Prior exposure to anti-TNF therapy, n (%)	61 (54.5)	46 (41.4)	32 (59.3)	72 (42.1)
Corticosteroid use, n (%)	47 (42.0)	37 (33.3)	32 (59.3)	52 (30.4)
Partial Mayo score	6.7 (1.2)	6.6 (1.4)	6.9 (1.4)	6.4 (1.4)

All values are mean (SD) unless otherwise stated.

Anti-TNF, anti–tumor necrosis factor; Bio-IR, inadequate response, loss of response, or intolerance to ≥1 biologic; FCP, fecal calprotectin; hsCRP, high-sensitivity C-reactive protein; RBS, rectal bleeding subscore; SD, standard deviation; SFS, stool frequency subscore.

a Among patients with mean daily SFS and RBS < 2.1 at maintenance week 0: an SFS and RBS each ≥1-point greater than the maintenance week 0 value on 2 consecutive visits ≥14 days apart. Among patients with SFS or RBS ≥ 2.1 at maintenance week 0: either an SFS or RBS ≥1-point greater than the maintenance week 0 value on 2 consecutive visits ≥14 days apart.

Patient characteristics were also balanced at induction baseline among those who experienced loss of response on UPA15 during maintenance vs those who did not (Table), with the following exceptions: patients with loss of response vs those without were more likely to have Bio-IR (61.1% vs 44.4%, respectively), prior exposure to anti-TNF therapy (59.3% vs 42.1%), or corticosteroid use at baseline (59.3% vs 30.4%).

### Efficacy

**Figure 2:**
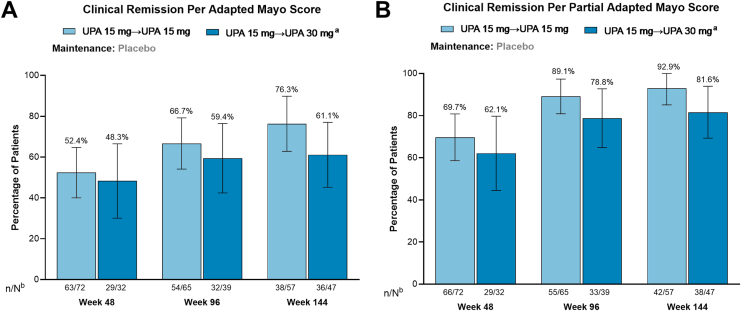
Proportion of patients in the UPA retreatment group who achieved clinical remission after 48, 96, and 144 weeks of the OLE study after temporary treatment interruption during the maintenance study. (A) Achievement of clinical remission per adapted Mayo score. (B) Achievement of clinical remission per partial adapted Mayo score. Error bars represent 95% confidence intervals. Clinical remission per adapted Mayo score is defined as an SFS ≤ 1 and not greater than baseline, RBS = 0, and endoscopy subscore ≤1 without friability. Clinical remission per partial adapted Mayo score is defined as an SFS ≤ 1 and RBS = 0. ^a^Patients with an inadequate response to UPA 15 mg, defined as an SFS + RBS that was unchanged or increased from week 0 on 2 consecutive visits ≥7 days apart, were escalated to UPA 30 mg. Patients had to escalate at least 12 weeks prior to the time point. ^b^N values are patient counts based on escalation status through week 36 for evaluation at week 48, week 84 for evaluation at week 96, and week 132 for evaluation at week 144. n, number of patients with available data; N, total number of patients on study treatment.

**Figure 3:**
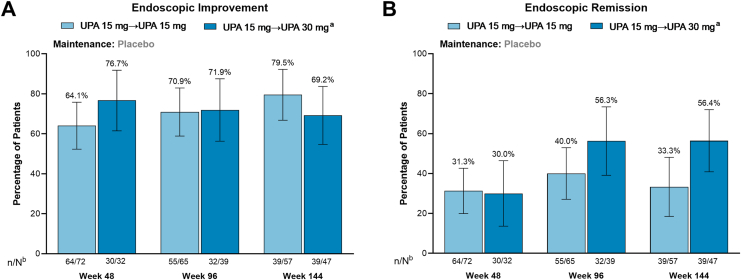
Proportion of patients in the upadacitinib retreatment group who achieved endoscopic improvement and endoscopic remission after 48, 96, and 144 weeks of the OLE study after temporary treatment interruption during the maintenance study. (A) Achievement of endoscopic improvement. (B) Achievement of endoscopic remission. Error bars represent 95% confidence intervals. Endoscopic improvement is defined as an endoscopic subscore of ≤ 1. Endoscopic remission is defined as an endoscopic subscore of 0. ^a^Patients with an inadequate response to UPA 15 mg, defined as an SFS + RBS that was unchanged or increased from week 0 on 2 consecutive visits ≥ 7 days apart, were escalated to UPA 30 mg. Patients had to escalate at least 12 weeks prior to the time point. ^b^N values are patient counts based on escalation status through week 36 for evaluation at week 48, week 84 for evaluation at week 96, and week 132 for evaluation at week 144. n, number of patients with available data; N, total number of patients on study treatment.

Among patients who entered the U-ACTIVATE OLE after losing response on placebo during the maintenance study, clinical remission per adapted Mayo score was achieved by 52.4% (number of patients achieving end point/number of patients with available data: 33/63), 66.7% (36/54), and 76.3% (29/38) of patients who remained on UPA 15 mg and 48.3% (14/29), 59.4% (19/32), and 61.1% (22/36) of patients who escalated to UPA 30 mg at weeks 48, 96, and 144, respectively (Figure 2A). Across all time points, most patients in the UPA retreatment groups also achieved clinical remission per partial adapted Mayo score (Figure 2B). Endoscopic improvement was achieved by 64.1%, 70.9%, and 79.5% of patients who remained on UPA 15 mg and 76.7%, 71.9%, and 69.2% of patients who escalated to UPA 30 mg at weeks 48, 96, and 144, respectively (Figure 3A). Endoscopic remission was achieved by 31.3%, 40.0%, and 33.3% of patients for those who remained on UPA 15 mg and 30.0%, 56.3%, and 56.4% of patients who escalated to UPA 30 mg at weeks 48, 96, and 144, respectively (Figure 3B).

**Figure 4:**
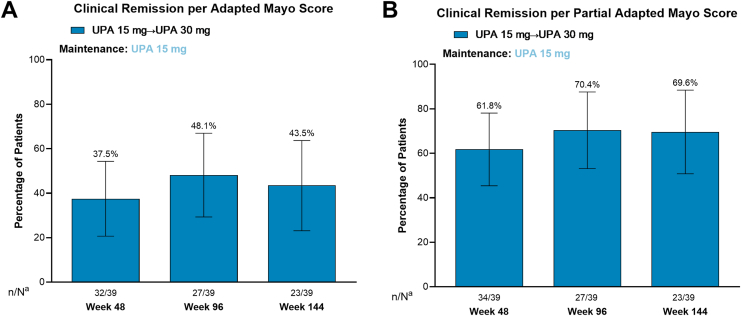
Proportion of patients in the UPA dose escalation group who achieved clinical remission after 48, 96, and 144 weeks of the OLE study after temporary treatment interruption during the maintenance study. (A) Achievement of clinical remission per adapted Mayo score. (B) Achievement of clinical remission per partial adapted Mayo score. Error bars represent 95% confidence intervals. Clinical remission per adapted Mayo score is defined as an SFS ≤ 1 and not greater than baseline, RBS = 0, and endoscopy subscore ≤ 1 without friability. Clinical remission per partial adapted Mayo score is defined as an SFS ≤ 1 and RBS = 0. ^a^N values are patient counts through week 36 for evaluation at week 48, week 84 for evaluation at week 96, and week 132 for evaluation at week 144. n, number of patients with available data; N, total number of patients on study treatment.

**Figure 5:**
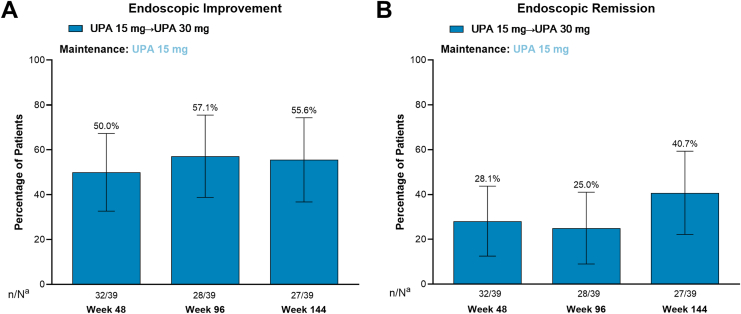
Proportion of patients in the UPA dose escalation group who achieved endoscopic improvement and endoscopic remission after 48, 96, and 144 weeks of the OLE study after temporary treatment interruption during the maintenance study. (A) Achievement of endoscopic improvement. (B) Achievement of endoscopic remission. Error bars represent 95% confidence intervals. Endoscopic improvement is defined as an endoscopic subscore of ≤ 1. Endoscopic remission is defined as an endoscopic subscore of 0. ^a^N values are patient counts through week 36 for evaluation at week 48, week 84 for evaluation at week 96, and week 132 for evaluation at week 144. n, number of patients with available data; N, total number of patients on study treatment.

Among patients who entered the OLE after losing response on UPA 15 mg during the maintenance study and were escalated to 30 mg after 4 weeks in the OLE (UPA15M→UPA30), clinical remission per adapted Mayo score was achieved by 37.5%, 48.1%, and 43.5% of patients at weeks 48, 96, and 144, respectively (Figure 4A). Most patients also achieved clinical remission per partial adapted Mayo score at all time points (Figure 4B). Endoscopic improvement was achieved by 50.0%, 57.1%, and 55.6% of patients at weeks 48, 96, and 144, respectively (Figure 5A). Endoscopic remission was achieved by 28.1%, 25.0%, and 40.7% of patients at weeks 48, 96, and 144, respectively (Figure 5B).

## Discussion

This analysis of the U-ACTIVATE OLE demonstrated that loss of clinical and endoscopic efficacy following withdrawal of the JAK inhibitor UPA or maintenance treatment on UPA 15 mg could be recaptured at week 48 upon UPA retreatment or dose escalation, respectively, and could be sustained through week 144.

A greater proportion of patients lost response when withdrawn from UPA during maintenance (50.2%; 112/223) vs those who lost response after receiving maintenance UPA 15 mg (24%; 54/225). This result underscores the risk associated with interrupting UPA treatment in terms of loss of efficacy.

In a real-world setting, patients with inflammatory bowel disease may consider treatment interruption or dose reduction to prevent or minimize the effects of comorbidities, reduce costs, and reduce the frequency of medication intake.^2^, ^3^, ^4^, ^5^, ^6^ The potential benefits from treatment interruption need to be weighed against the significant risks that are involved.^2^^,^^4^, ^5^, ^6^ This study demonstrates that an induction-only treatment strategy can lead to loss of efficacy, as approximately half of patients who were withdrawn from UPA lost response. Retreatment with UPA15 could recapture efficacy end points at week 144 (approximately 3 years) in 54.8% (57/104) of these patients; however, 45.2% (47/104) required dose escalation. Dose escalation following loss of response on maintenance UPA15 could also recapture efficacy end points in 40.7%–69.6% of patients, depending on the end point.

Although safety was not assessed in this analysis of the U-ACTIVATE OLE, an interim analysis of U-ACTIVATE demonstrated that UPA was well-tolerated over 96 weeks in the OLE, with only a minority of treatment-emergent adverse events classified as serious, rates of adverse events of special interest in line with previous UPA studies, and rare occurrences of adjudicated major adverse cardiovascular events, venous thromboembolisms, and malignancies, also in line with previous UPA studies.^13^

A previous study of the JAK inhibitor tofacitinib in patients with UC also demonstrated that efficacy can be recaptured following retreatment.^14^ When taken together with this analysis, these results suggest an ability for JAK inhibitors to block the inflammatory pathways driving disease pathogenesis following a period of reactivation resulting from treatment cessation.

In this analysis, clinical and endoscopic efficacy were largely, but not entirely, recaptured in both the UPA retreatment and dose escalation populations. These findings suggest patient-level variations in response to UPA ranging from maintaining response even after treatment withdrawal to the inability to recapture response following extended retreatment. Approximately half of patients did not lose response when placed on placebo during the maintenance study after responding to UPA induction (111/223, 49.8%). Conversely, a subset of patients in the UPA retreatment group did not achieve clinical remission even after retreatment with UPA15 and dose escalation to UPA30. Future studies are required to identify novel predictive factors related to responsiveness of retreatment, such as clinical and pharmacodynamic biomarkers in patients in whom efficacy was not recaptured. This may help health-care providers make informed decisions regarding UPA treatment discontinuation and subsequent retreatment or escalation.

Key limitations to this study include the limited number of patients, particularly in the dose escalation population; therefore, the results should be interpreted with caution. Additionally, only descriptive statistical analyses were performed. This study did not evaluate efficacy recapture in patients who lost response when de-escalating from a 45 mg dose of UPA down to 30 mg. This study also did not evaluate efficacy recapture following retreatment with a 45 mg dose of UPA as reinduction following treatment withdrawal, which may occur in a real-world setting. Further studies in a real-world setting with larger populations will advance our understanding of how treatment de-escalation followed by retreatment or dose escalation impacts efficacy of UPA and possibly predict patients in whom these scenarios would recapture efficacy.

## Conclusion

This analysis of the U-ACTIVATE OLE suggests that UPA therapy may successfully recapture efficacy following treatment de-escalation. Longer studies are required to assess the safety profile of patients who restart treatment or dose escalate following a loss of efficacy. Future investigation into predictors influencing successful efficacy recapture is also required to fully inform the decision to de-escalate treatment.

## Supplementary Materials

Supplementary Appendix

Extended PDF

## References

[1] Glasziou P., Irwig L., Mant D. (2005). Monitoring in chronic disease: a rational approach. BMJ.

[2] Israel A., Jurdi K.E., Rubin D.T. (2019). Treatment de-escalation in patients with inflammatory bowel disease. Gastroenterol Hepatol (N Y).

[3] Miyatani Y., Kobayashi T. (2023). De-escalation of therapy in patients with quiescent inflammatory bowel disease. Gut Liver.

[4] Ungaro R.C. (2022). De-escalation of therapy for patients with inflammatory bowel disease. Gastroenterol Hepatol.

[5] Rubin D.T. (2019). Restarting biologic agents after a drug holiday. Gastroenterol Hepatol (N Y).

[6] St-Pierre J., Shafrir A., Rubin D.T. (2024). Interrupting inflammatory bowel disease therapy: why, who, when and how to consider medication holidays. Expert Rev Gastroenterol Hepatol.

[7] Danese S., Vermeire S., Zhou W. (2022). Upadacitinib as induction and maintenance therapy for moderately to severely active ulcerative colitis: results from three phase 3, multicentre, double-blind, randomised trials. Lancet.

[8] Vermeire S., Danese S., Zhou W. (2023). Efficacy and safety of upadacitinib maintenance therapy for moderately to severely active ulcerative colitis in patients responding to 8 week induction therapy (U-ACHIEVE Maintenance): overall results from the randomised, placebo-controlled, double-blind, phase 3 maintenance study. Lancet Gastroenterol Hepatol.

[9] RINVOQ (upadacitinib) [prescribing information]. https://www.rxabbvie.com/pdf/rinvoq_pi.pdf.

[10] Turner D., Ricciuto A., Lewis A. (2021). STRIDE-II: an update on the Selecting Therapeutic Targets in Inflammatory Bowel Disease (STRIDE) initiative of the International Organization for the Study of IBD (IOIBD): determining therapeutic goals for treat-to-target strategies in IBD. Gastroenterology.

[11] Panaccione R., Danese S., Zhou W. (2024). Efficacy and safety of upadacitinib for 16-week extended induction and 52-week maintenance therapy in patients with moderately to severely active ulcerative colitis. Aliment Pharmacol Ther.

[12] Sandborn W.J., Ghosh S., Panes J. (2020). Efficacy of upadacitinib in a randomized trial of patients with active ulcerative colitis. Gastroenterology.

[13] Panaccione R., Vermeire S., Danese S. (2025). Long-term efficacy and safety of upadacitinib in patients with moderately to severely active ulcerative colitis: an interim analysis of the phase 3 U-ACTIVATE long-term extension study. Lancet Gastroenterol Hepatol.

[14] Panés J., Vermeire S., Dubinsky M.C. (2021). Efficacy and safety of tofacitinib re-treatment for ulcerative colitis after treatment interruption: results from the OCTAVE clinical trials. J Crohns Colitis.

