# Supplementary material for: Efficacy of Upadacitinib Retreatment or Dose Escalation After Loss of Response in Ulcerative Colitis: Data From the Open-Label Extension of the U-ACTIVATE Study
Source: Gastro Hep Adv. 2026 Apr 13;5(7):100969. doi: 10.1016/j.gastha.2026.100969 (PMC13235344; doi:10.1016/j.gastha.2026.100969)
Supplement: Supplementary Appendix [file mmc1.pdf]

Supplementary Appendix.

Efficacy of Upadacitinib Retreatment or Dose Escalation After Loss of Response in Ulcerative Colitis: Data From the Open-Label Extension of the U-ACTIVATE study

Running Title: Upadacitinib Retreatment Efficacy Recapture

Remo Panaccione,[1] Jean-Frédéric Colombel,[2] Marla Dubinsky,[2] Christopher Ma,[1] Michelle Kujawski,[3] Erica Cheng,[3] Elena Dubcenco,[3] Sina Ogholikhan,[3] Elena Marced Barrachina,[3] Tadakazu Hisamatsu[4]

[1]Division of Gastroenterology and Hepatology, University of Calgary, Calgary, AB, Canada.

[2]Division of Gastroenterology and Hepatology, Icahn School of Medicine at Mount Sinai, New York, NY, USA.

[3]AbbVie Inc., North Chicago, IL, USA.

[4]Department of Gastroenterology and Hepatology, Kyorin University School of Medicine, Tokyo, Japan.

Supplemental Figure S1. Number of Patients Stratified by UPA Dose During the U-ACTIVATE OLE Among Patients Who Lost Response to Placebo During Maintenance

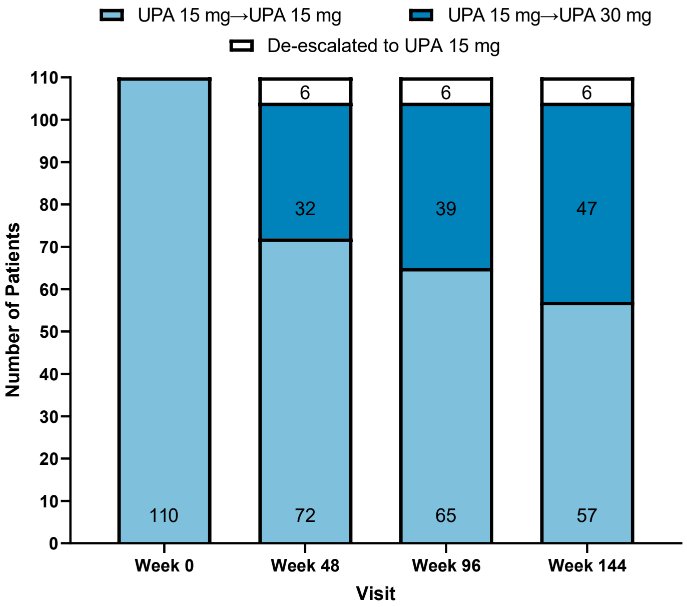

Abbreviations: OLE, open-label extension; UPA, upadacitinib.

Supplemental Table 1. Patient Characteristics at Week 144 Among Those Who Did Not Undergo Dose Escalation (UPA15→UPA15) and Those Who Underwent Dose Escalation (UPA15→UPA30) During the U-ACTIVATE OLE.

	Lost Response on Maintenance: Placebo[a] (n = 110)	
	**UPA15→UPA15 (n = 57)**	**UPA15→UPA30 (n = 53)**
**Female, n (%)**	23 (40.4)	23 (43.4)
**Age, years**	41.4 (14.8)	42.5 (14.2)
**Race, n (%)**		
Asian	24 (42.1)	11 (20.8)
White	31 (54.4)	39 (73.6)
Other	2 (3.5)	3 (5.7)
**Disease duration, years**	8.6 (7.6)	7.0 (6.4)
**Disease extent, n (%)**		
Rectosigmoid	0	0
L-sided	27 (47.4)	33 (62.3)
Pancolitis	30 (52.6)	20 (37.7)
**FCP, mg/L**	3147 (5182)	3550 (5329)

**hsCRP, mg/L**	6.6 (10.9)	12.1 (20.2)
**Bio-IR, n (%)**	28 (49.1)	35 (66.0)
**Corticosteroid use, n (%)**	20 (35.1)	26 (49.1)
**Prior exposure to biologic therapy, n (%)**	0	3 (16.7)
**Prior exposure to anti-TNF therapy, n (%)**	24 (42.1)	36 (67.9)
**Partial Mayo score**	6.7 (1.2)	6.7 (1.3)

Abbreviations: anti-TNF, anti-tumor necrosis factor; Bio-IR, inadequate response, loss of response, or intolerance to ≥ 1 biologic; FCP, fecal calprotectin; hsCRP, high-sensitivity C-reactive protein; OLE, open-label extension; RBS, rectal bleeding subscore; SD, standard deviation; SFS, stool frequency subscore; UPA, upadacitinib.

All values are mean (SD) unless otherwise stated.

[a]Among patients with mean daily SFS and RBS < 2.1 at maintenance week 0: an SFS and RBS each ≥ 1-point greater than the maintenance week 0 value on 2 consecutive visits ≥ 14 days apart. Among patients with SFS or RBS ≥ 2.1 at maintenance week 0: either an SFS or RBS ≥ 1-point greater than the maintenance week 0 value on 2 consecutive visits ≥ 14 days apart.
